# A Low-Cost Imaging Method for the Temporal and Spatial Colorimetric Detection of Free Amines on Maize Root Surfaces

**DOI:** 10.3389/fpls.2017.01513

**Published:** 2017-08-30

**Authors:** Truc H. Doan, Tu A. Doan, Michael J. Kangas, AdreAnna E. Ernest, Danny Tran, Christina L. Wilson, Andrea E. Holmes, Erin L. Doyle, Tessa L. Durham Brooks

**Affiliations:** ^1^Department of Biology, Doane University, Crete NE, United States; ^2^Department of Chemistry, Doane University, Crete NE, United States; ^3^Crete High School, Crete NE, United States

**Keywords:** rhizosphere, root exudates, maize seedling, colorimetric detection, ninhydrin, printing sensors, root imaging

## Abstract

Plant root exudates are important mediators in the interactions that occur between plants and microorganisms in the soil, yet much remains to be learned about spatial and temporal variation in their production. This work outlines a method utilizing a novel colorimetric paper to detect spatial and temporal changes in the production of nitrogen-containing compounds on the root surface. While existing methods have made it possible to conduct detailed analysis of root exudate composition, relatively less is known about where in the root system exudates are produced and how this localization changes as the root grows. Furthermore, there is much to learn about how exudate localization and composition varies in response to stress. Root exudates are chemically diverse secretions composed of organic acids, amino acids, proteins, sugars, and other metabolites. The sensor utilized for the method, ninhydrin, is a colorless substance in solution that reacts with free amino groups to form a purple dye. A detection paper was developed by formulating ninhydrin into a print solution that was uniformly deposited onto paper with a commercial ink jet printer. This “ninhydrin paper” was used to analyze the chemical makeup of root surfaces from maize seedlings grown vertically on germination paper. Through contact between the ninhydrin paper and seedling root surfaces, combined with images of both the seedlings and dried ninhydrin papers captured using a standard flatbed scanner, nitrogen-containing substances on the root surface can be localized and concentration of signal estimated for over 2 weeks of development. The method was found to be non-inhibiting to plant growth over the analysis period although damage to root hairs was observed. The method is sensitive in the detection of free amines at concentrations as little as 140 μM. Furthermore, ninhydrin paper is stable, showing consistent color changes up to 2 weeks after printing. This relatively simple, low-cost method could contribute to a better understanding of root exudates and mechanisms used by plants to interact with the complex soil environment during growth and development.

## Introduction

The rhizosphere is a complex and dynamic microecosystem formed between plant roots and their associated microbes, single-celled eukaryotes, mycorrhizal fungi, and small soil-dwelling multicellular organisms (Badri and Vivanco, 2009). At its core, it is a partnership between roots, which exude a variety of metabolites, ions, and cofactors, and microbes that respond to, utilize, and process them (Badri and Vivanco, 2009; Dennis et al., 2010; Kumar et al., 2012). Interactions of exudates with microbes in the rhizosphere can have significant benefits to the plant including mobilization of soil nutrients, prevention of and enhanced response to infection, and promotion of plant growth and development (Badri and Vivanco, 2009; Kumar et al., 2012). Advances in DNA sequencing technology have shown that microbial composition and abundance of the rhizosphere differs from that of the bulk soil, as evidenced by a decline in diversity and an increase in specialization near the root surface (Micallef et al., 2009; Bulgarelli et al., 2013; Peiffer et al., 2013). Root exudates are thought to be a primary driver of microbial community makeup (Micallef et al., 2009; Dennis et al., 2010; Bulgarelli et al., 2013). Additionally, root exudates play a critical role in plant–plant communication and mediate a variety of positive and negative interactions between nearby plants such as the induction of defense responses (Walker et al., 2003). Therefore, understanding exudate composition and determining genetic and environmental factors that influence its production is a primary focus of crop improvement efforts (Bulgarelli et al., 2013; Chaparro et al., 2013).

Root exudates represent a significant portion (20–50%) of the total metabolism of the plant (Aguiar et al., 2014). They are produced within the root and transported into the nearby soil environment by root epidermal cells and root hairs, and by the production of mucilage and the sloughing off of cells at the root cap (Walker et al., 2003; Dennis et al., 2010). Root exudates are composed of a mix of primary metabolites including sugars, amino acids and organic acids. In smaller abundance are fatty acids, sterols, cofactors, and regulatory molecules (Aguiar et al., 2014). Their composition changes with the plant’s genetic makeup, development of the plant and in response to stress. Both biotic and abiotic stressors are able to mediate both positive and negative interactions (via exudate components) between rhizosphere microbes and surrounding plants (Czarnota et al., 2001; Walker et al., 2003; Warembourg et al., 2003; Micallef et al., 2009; Dennis et al., 2010). Amino acids represent the second-most abundant component of root exudates (Chaparro et al., 2013; Moe, 2013). They contribute to nitrogen cycling in the soil and are important in attracting beneficial root microbes (Jones et al., 2002; Oku et al., 2012; Moe, 2013). The amino acid exudate gamma-aminobutyric acid can act as a signal to prime root defenses (Jakab et al., 2001; Moe, 2013). Finally, amino acid exudates, particularly tryptophan, can be important signals and metabolic intermediates affecting root growth and development through the production of phytoactive hormones by plant growth promoting bacteria (PGPB) (Sukumar et al., 2013; Koul et al., 2015).

Maize exudates contain a variety of free-amine containing compounds, primarily in the form of amino acids (Kraffczyk et al., 1984; Baudoin et al., 2003; Fischer et al., 2010). Glutamate is thought to be a primary component of this portion of the exudate, though serine, alanine and glycine have also been observed in relatively high proportions (Baudoin et al., 2003; Fischer et al., 2010) and its concentration increases with the presence of growth-promoting rhizobacteria and certain nutrient deficiencies (Kraffczyk et al., 1984; Carvalhais et al., 2011). Functionally, maize root exudates play roles similar to what they do in other species including nutrient mobilization, enhanced defense response, and attraction of growth-promoting bacteria (Mench and Martin, 1991; Baetz and Martinoia, 2014; Zhang et al., 2015).

Only recently have metabolomic studies been undertaken to more systematically describe the chemical makeup of exudates (Chaparro et al., 2013). While it is known that apical structures of the root are generally more active in producing exudates, the spatial distribution of exudates and how they change with time remain largely unexplored (Walker et al., 2003; Bulgarelli et al., 2013). Understanding the complex relationships between plant roots, root exudates, and rhizosphere microbes requires detailed measurements of exudate composition and their spatial/temporal distribution. A number of approaches have been published, all of which have advantages and costs. Root exudates may be collected from roots grown in soil, hydroponically, or on other solid support systems such as glass beads, nutrient agar or a capillary mat (Czarnota et al., 2001; Ernst et al., 2010; Dundek et al., 2011; Chaparro et al., 2013; Aguiar et al., 2014; Slota et al., 2016). For some exudate collection methods, roots are washed to remove residual soil, nutrients, and dead cells and are then incubated in a bath solution from which the exudates are recovered (Dundek et al., 2011). This approach, while providing a more natural growing environment, has the disadvantage of being destructive, thereby only allowing a single time point to be collected for each plant. Some experimental setups allow for exudates to be non-destructively sampled as hydroponic media is continuously perfused through growth system and collected, but localization of exudate production in these systems is difficult (Oburger et al., 2013; Slota et al., 2016). Another way to track exudates is to introduce radioactive isotopes in combination with imaging techniques (Oburger and Schmidt, 2016), but introduction of radioisotopes into the lab is not feasible in some research settings.

Water-soluble exudates are typically filtered and concentrated prior to analysis (Dundek et al., 2011). The recovered exudates may be fractionated using chromatography techniques and sample fractions are characterized using UV-vis, mass spectrometry, or NMR (Micallef et al., 2009; Kim et al., 2010; Dundek et al., 2011; Aguiar et al., 2014). In a targeted analysis approach known exudate compounds are detected in the sample. However, methods for non-targeted profiling of exudate composition are rapidly growing and can lead to discovery of new compounds in the exudate and determinations of how exudate composition changes with a given condition (Marti et al., 2013; Oburger et al., 2013). Any metabolomics approach necessarily requires use of highly specialized equipment. A much more accessible approach has been to use colorimetric indicators and assays to more generally assess total carbohydrate, sugar, or protein content (Van Egeraat, 1975; Dundek et al., 2011). Intact roots can also be exposed to a variety of indicators to detect the location of production and release of specific molecules. For example, blotting exposed plant roots with agar sheets containing the indicators bromocresol purple or BPDS were used to localize production of protons or iron-reducing compounds, respectively (Marschner et al., 1982; Römheld et al., 1984; Neumann et al., 2009). However, the agar sheets must be carefully prepared and stored, which adds difficulty and tedium to the procedure. In summary, there have been a variety of methods developed for collecting, detecting and analyze root exudate, but a need remains for development of simple (and higher throughput) approaches to enable spatial and temporal descriptions of exudate production.

**FIGURE 1:**
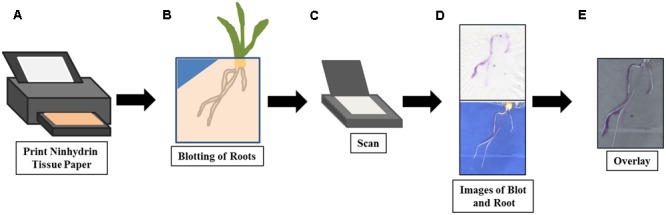
Method overview for root exudate imaging with ninhydrin. The ninhydrin paper was printed **(A)** with 3% ninhydrin ink solution and allowed to dry. On data acquisition days, the maize root was blotted gently **(B)** with ninhydrin paper to absorb the exudates released by the root. After the blotted ninhydrin paper was allowed to develop, it and the root were scanned **(C)** using a flatbed scanner and images collected **(D)**. Finally, the ninhydrin paper and seedling images were overlaid **(E)** to visualize localization of exudates.

We have developed a simple, minimally disruptive method for detecting and measuring root exudate production on the surface of a growing root (**Figure 1**). The colorimetric indicator ninhydrin has been used to detect free amine groups in exudates on the root surface. Ninhydrin is a useful compound for this purpose due to the easily visualized purple-colored product that is formed in the presence of analyte. Ninhydrin is also sensitive to free amines at physiological concentrations (Van Egeraat, 1975; Dundek et al., 2011; Chutipongtanate et al., 2012). A novel process for printing the indicator on commercially available tissue paper was developed and roots grown vertically on germination paper were blotted with this paper to capture a snapshot of free-amines on the root surface. Images of blotted ninhydrin papers and seedlings were captured and image processing tools developed to visualize free amine localization and relative concentration. Because our method does not disturb the plant as is grows and the indicator formulation is non-toxic to the seedlings, it can be used to collect time course data for individual plants. Additionally, we have determined that the ninhydrin-printed paper is shelf-stable and reliably detects root exudates at biologically relevant concentrations. Here, we demonstrate the application of our method to maize roots, but it could readily be expanded to other plant systems and indicators.

## Materials and Methods

### Preparation of Ninhydrin Paper

All reagents and chemical products were purchased from Sigma–Aldrich (St. Louis, MO, United States) at technical grade or better and were used without further purification. The print solution was composed of 71.2% (v/v) sodium acetate buffer (0.1 mM, pH 5), 14.8% glycerol, 8.2% diethylene glycol, and 5.8 % of triethylene glycol monobutyl ether.

Ninhydrin ink (3% w/v) was prepared using the print solution as the solvent and sonicated (Bransonic ultrasonic bath, CPX2800H) for 3 h at room temperature. The solution was stored in an amber bottle in a dark cabinet at room temperature.

The 3% ninhydrin ink was added to an empty cartridge (InkOwl, Champlain, NY, United States) using a syringe (with 0.8 μm filter) and placed into the printer (Canon MG5520). The high quality setting on the printer was used. Commercially available white tissue paper (Hallmark, Kansas City, MO, United States) cut to size (8.5 in × 12 in) was taped onto regular copy paper using all-purpose labels (Avery, 6737) to facilitate feeding of the tissue paper through the printer. The template provided in **Supplementary Presentation S1** was used to uniformly print ninhydrin on the paper, except for two 3 × 3 grids used for paper calibration and quantification of exudates. After printing, the ninhydrin paper was placed in an opaque envelope and stored at ambient temperature and humidity until used.

### Preparation of Mock Exudates

A mock exudate solution was prepared according to Baudoin et al. (2003) for use in determining ninhydrin paper sensitivity and longevity. The 70X mock exudate consisted of 18.4 mM glucose, 18.4 mM fructose, 9.2 mM sucrose, 9.2 mM citric acid, 18.4 mM lactic acid, 13.8 mM succinic acid, 9.2 mM alanine, 9.2 mM serine, and 5.5 mM glutamic acid. The total free amine concentration of 70X mock exudate was 23.9 mM. When diluted to 1X with distilled water, carbon content of mock exudate was at a concentration similar to previously published values (Iijima et al., 2000) and the C/N ratio was similar to previously reported values (Kraffczyk et al., 1984).

### Germination and Planting

Seeds of maize inbred genotype B73 were sterilized by washing with 70% ethanol for 3 min and 2% (w/v) bleach for 5 min (Naveed et al., 2014). The seeds were then washed three times with autoclaved distilled water, stirring for 1 min for each wash. The seeds were rolled in paper towels wetted with sterile distilled water. The paper rolls were then covered by aluminum foil and imbibed at 4°C for 2 days (due to improved germination of B73 during cold imbibition) then moved to a growth chamber (Percival, Geneva Scientific) set at 24°C with continuous light (to avoid circadian effects) and with a 75% relative humidity (van de Venter and Grobbelaar, 1985; Naveed et al., 2014).

Three-day old seedlings were planted using a pouch method (Hund et al., 2009). Blue germination paper (Anchor Paper Co.) was trimmed to 8.5 in × 12 in, and a small triangle was removed from the center of the top edge. Seedlings were positioned at the apex of the triangle. Polyethylene sheets were cut approximately to the size and shape of the germination paper and positioned over it. Seedlings were held to the germination paper by placing paper clips on each side of the triangle apex. Plastic sheets and blotter germination paper were clipped to a wooden dowel using two binder clips and the dowel was used to hang the entire seedling apparatus using either end of the rim of 8-gallon opaque plastic containers (**Supplementary Figure S1**). Each 8-gallon containers held up to ten seedlings and were filled with enough sterilized distilled water to reach the bottom of the germination paper. Lightweight black cloth was draped over the either side of the seedling to keep the roots relatively dark. Seedlings were grown at 24°C with continuous light and 75% relative humidity.

### Blotting and Scanning

Pouches were removed from the growth chamber every 2 days for scanning and blotting. The polyethylene sheet covering the surface of the root was pulled back to expose the root surface for blotting. To minimize water contacting and wrinkling the ninhydrin paper during blotting, laminated paper was used to cover portions of the germination paper where the root had not grown. The ninhydrin paper was gently pressed to the root for approximately 10 s (**Supplementary Figure S2**). After blotting, the ninhydrin paper was dried for approximately 24 h at room temperature with compression in a press (Carolina, Cat #: 663050) to eliminate wrinkles in the paper during scanning. The blotted ninhydrin papers were placed on the flatbed surface and color images were acquired using an EPSON V700 scanner and VueScan x64 software with settings shown in Supplementary Table S1.

An EPSON V700 scanner using VueScan x64 software with settings shown in Supplementary Table S2 was used for collecting RGB images of the roots. To minimize bubbles on the blue blotter germination paper and to ensure a consistent background, a clear plastic sheet was placed on the surface of the scanner, and the surface of the scanner was flooded with a small volume of water that was contained within the beveled edge of the scanner housing. The plastic sheet covering the root was lifted off the root surface and the seedling pouch was carefully placed onto the flooded scanner surface with the seedling facing down. Pouches were oriented in the same manner as the ninhydrin papers.

### Image Analysis

ImageJ software, which is freely available, open-source, and able to run on nearly any operating system, was used for analysis of spot intensities and root lengths (Schneider et al., 2008). Primary root length was measured using the segmented line tool. For determining paper sensitivity and stability, average pixel intensity of a spot created by depositing 1 μL of mock exudate on ninhydrin papers was determined by visually selecting areas containing signal with the magic wand tool. The average pixel intensity was quantified using the RGB measure function. Average pixel intensity was calculated as the weighted sum of the red, green and blue channels (0.30 R + 0.59 G + 0.11 B). This method can be set as an option in ImageJ (Edit/Options/Conversions menu).

The program GIMP2 (GNU Image Manipulation Program)^1^ was used to produce overlay images between signals in a ninhydrin paper and seedling roots growing on germination paper. After opening the seedling root image it was converted to grayscale using the Colors/Desaturate command. The ninhydrin paper image was imported by dragging it onto the seedling image or by using File/‘Open as Layers’ from the seedling image. The ninhydrin paper layer was overlaid using a multiply operation. The ‘Mulitply’ option was selected from the ‘Mode’ pull-down menu in the ‘Layers’ toolbox to produce the overlay. The orientation of the ninhydrin paper was adjusted using the Move Tool as needed. The image overlay was exported in the desired format using the File/Export command.

### Determination of Seedling Growth Rate

Seedling growth rate (*R*) for a given day (*D*) was determined using the following formula where (L) is length in cm:

(1) $$\begin{array}{c} R_{D}=\frac{L_{D+2}-L_{D}}{2} \end{array}$$

### Paper Calibration

To calibrate ninhydrin papers for root exudates quantification, three replicate 0.1 μL aliquots of mock exudate containing free amine concentrations of 1.71, 3.41, or 4.71 μM were deposited on one of the two printed grids on the paper immediately prior to time of blotting. After a 24 h incubation period in a press and imaging the paper as described above, the magic wand tool (ImageJ) was used to manually capture the developed areas for each aliquot. The average pixel intensity of each calibration spot was calculated in ImageJ as described above. For each of the three mock exudate concentrations, the lower and upper quartiles (Q3 and Q1) and the interquartile range (IQR, calculated as Q3-Q1) for average pixel intensity were calculated using the calibration spots of that concentration from the blotted ninhydrin papers for 3-, 5-, and 7-day old seedlings. An individual calibration spot was considered to be an outlier if its average pixel intensity was less than Q1 – 0.5^∗^IQR or greater than Q3 + 0.5^∗^IQR for the concentration. Papers with two or more outlier calibration spots for a single mock exudate concentration were determined to be outlier papers and were removed from the dataset.

For the retained papers, a linear regression was used to model the relationship between the known concentration of mock exudate applied and the measured pixel intensity of each spot. The regression equation is shown below, where y is the average pixel intensity and x is the free amine concentration in the mock exudates (*R*^2^ = 0.72, *p* = 0.0):

(2) $$\begin{array}{c} y\;=\;-20.98x\;+\;241.71 \end{array}$$

A 95% confidence interval (CI) was calculated for the regression line to determine the range of concentrations expected based on given average pixel intensity values.

In order to visualize exudate concentrations on the root surface, the blotted and scanned ninhydrin paper images were read using the Python OpenCV library and converted to gray-scale. Using the regression equation and computed CI, pixel intensities across the entire image were converted to estimated free amine concentrations. A heat map function was applied to re-draw the image with approximate exudate concentrations indicated by a color gradient. A custom Python script for executing this visualization is provided as **Supplementary Data Sheet S1**.

## Results

### Method Overview

The method workflow is illustrated in **Figure 1**. Seedlings are grown using a modified pouch method, which allows easy access and visualization of the root surface (Hund et al., 2009). The roots are gently pressed against paper printed with a ninhydrin solution (**Figures 1A,B**). The blotted ninhydrin paper and the seedling roots are scanned and the resulting digital images overlaid to identify regions of the root that are active in nitrogenous exudate production (**Figures 1D,E**). After calculating the average pixel intensities of spots containing known free amine concentrations, outlier papers can be identified and free-amine containing exudate concentration in the remaining papers can be estimated (not shown).

### Ninhydrin Paper Sensitivity to Free Amines

**FIGURE 2:**
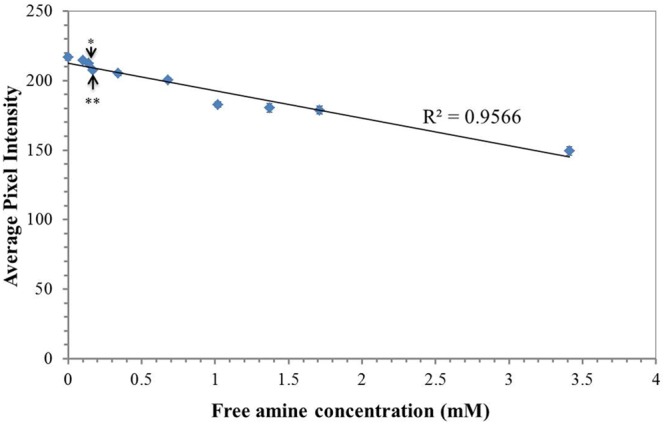
Paper sensitivity for free amines. Average pixel intensity (0.30 R + 0.59 G + 0.11 B) of selected regions of 3% ninhydrin paper spotted with 1 μL of a solution containing various concentrations of free amines in mock exudates. Points are the mean (*n* = 36, 4 sheets, 9 spots measured per sheet) ± standard error. ^∗∗^ indicates a statistically significant difference (*p* < 0.01) between each sampling concentration to the control (water, 0 μM free amine). Statistical significance was determined using a two-way ANOVA with exudate free-amine concentration and paper (*n* = 4) as the factors and average intensity as the dependent variable. Pairwise comparisons were made from a *post hoc* Tukey’s HSD. All points more concentrated than the free amine concentration indicated by the double asterisks were significantly different from the control (*p* < 0.01).

The sensitivity of ninhydrin paper to the detection of free amines was determined by measuring the average pixel intensity of selected regions of 3% ninhydrin paper spotted with 1 μL of mock exudate solutions containing known free amine concentrations (**Figure 2**). Free amine concentrations between 0 and 3.41 mM were tested. A grid of nine 1 μL volumes for each mock exudate concentration was deposited onto on four separate ninhydrin papers. The aliquoted volumes were allowed to spread, creating uniform spots on the paper. Papers were stored in an opaque envelope in the dark overnight in order to develop the indicator. The colorimetric transition from white/pale yellow to purple was apparent within this time frame. Papers were scanned and average pixel intensity determined using ImageJ. Free amine concentrations as little as 140 μM were detected based on statistically significant differences compared to the control (*p* < 0.01, two-way ANOVA, Tukey *post hoc*). All concentrations tested were within the linear range of the indicator based on the linear trend of indicator response with concentration (**Figure 2**) and within the physiological range of the amine content expected in the exudate (Baudoin et al., 2003). Therefore, ninhydrin paper is sensitive to exudates within the physiological range.

### Ninhydrin Paper Stability in Atmospheric Conditions

**FIGURE 3:**
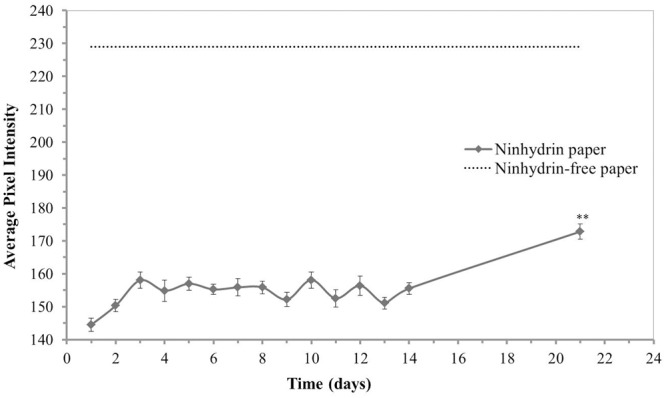
Stability of ninhydrin paper. Average pixel intensity (0.30 R + 0.59 G + 0.11 B) of selected regions of 3% ninhydrin paper spotted with 1 μL of a mock exudate solution containing 3.41 mM free amines then developed overnight. Values presented are means ± standard error (*n* = 27, 3 sheets, 9 spots measured per sheet). *Post hoc* analysis (Tukey’s HSD) of a two-way ANOVA with paper (*n* = 3) and mock exudate free-amine concentration as factors and pixel intensity as the dependent variable determined that day 21 intensities were significantly different from every other day (*p* < 0.01).

The optimal use period, or time from printing until which a stable signal could be achieved, was determined for conditions typical to the laboratory (room temperature and ambient humidity). Ninhydrin paper was treated with mock exudate containing 3.41 mM free amines every day for 21 days after printing with 3% ninhydrin print solution (**Figure 3**). The ninhydrin paper was stored in an opaque envelope to protect it from light. The average pixel intensity was found to change most dramatically the first day after printing as observed by the increase from 134.77 ± 2.63 to 147.29 ± 3.09. This is hypothesized to be the result of the ninhydrin ink drying which at atmospheric humidity and temperature takes at least 2 days to complete. On day three to fourteen the RGB intensity stabilized to an average value of 150 with insignificant fluctuation in signal during this period (*p* > 0.05, two-way ANOVA, Tukey *post hoc*). After 21 days in storage a significant drop in ninhydrin paper sensitivity was observed which is hypothesized to stem from interaction of the paper with humid air or light, which could likely be mitigated by storage of the paper under more controlled conditions. Based on these data, ninhydrin paper was conservatively deemed optimal for blotting 4–13 days after printing.

### Seedling Sensitivity to Ninhydrin Paper

**FIGURE 4:**
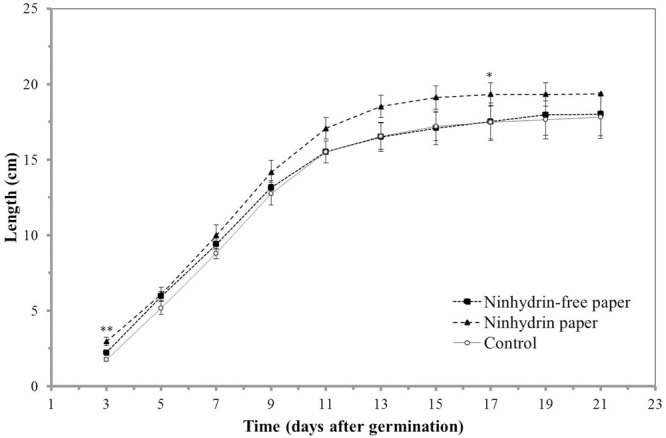
Impact of ninhydrin paper blotting on root length. Growth of B73 seedling roots along the germination paper surface without blotting (Control), while blotting seedlings with plain paper every 2 days (Ninhydrin-free paper), or while blotting with 3% ninhydrin paper every 2 days (Ninhydrin paper). Germination occurred at day 0. The data are the mean of 10 seeds for control, 9 seeds for the ninhydrin-free paper control, and 10 seeds for ninhydrin paper. Error bars are the standard error of the mean. ^∗^ indicates a statistically significant difference (*p* < 0.05) and ^∗∗^ indicates a statistically significant different (*p* < 0.01) between the ninhydrin paper condition and the control as determined by a Student’s *t*-test. Note that seedlings blotted with ninhydrin paper were slightly, but significantly longer than roots used for the other two groups at the start of the experiment.

**FIGURE 5:**
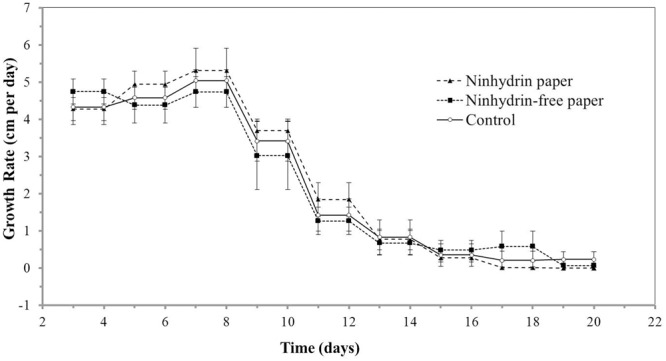
Growth rates of roots exposed to ninhydrin paper. Growth rate (cm/day) of B73 seedling roots along the germination paper surface unperturbed without ninhydrin (Control), while blotting with plain paper every 2 days (Ninhydrin-free paper), or while blotting with paper containing 3% ninhydrin every 2 days (Ninhydrin paper). Germination occurred at day 0. The data are the mean of 10 seeds for control, 9 seeds for the blotting control, and 10 seeds for 3% ninhydrin. Error bars are the standard error of the mean.

As root growth is sensitive to environmental factors, such as chemical and mechanical stress, the impact of blotting with ninhydrin paper on root growth was determined. Insignificant differences were observed in growth length (**Figure 4**) and growth rate (**Figure 5**) between roots that were not blotted (Control), roots blotted with ninhydrin-free paper, and roots blotted with 3% ninhydrin paper. Seedlings that were blotted with ninhydrin paper were slightly, but significantly longer than roots used for the other two groups at the start of the experiment (**Figure 4**, *p* < 0.01, Student’s *T*-test). Ninhydrin-free paper was used to determine the impact of mechanical stress of blotting while use of 3% ninhydrin paper indicates the potential impact of mechanical stress as well as chemical stress should ninhydrin be transferred from the paper to the root during contact. Roots achieved linear growth rates from day three through nine and plateaued in growth at around 15–18 cm after experimental day thirteen (**Figure 4**). The trend in growth rate was similar in all treatment groups (**Figure 5**) with roots growing at a steady rate of 4.5 cm/day from days three through eight of blotting followed by incremental decreases to 3.3, 1.2, 0.5, and 0.25 cm per day days 9, 11, 13, and 15 respectively. Overall, growth length and rate remained unaffected by mechanical stress and roots did not show any indication of toxicity to ninhydrin exposure.

### Root Hair Growth and Exudate Localization

**FIGURE 6:**
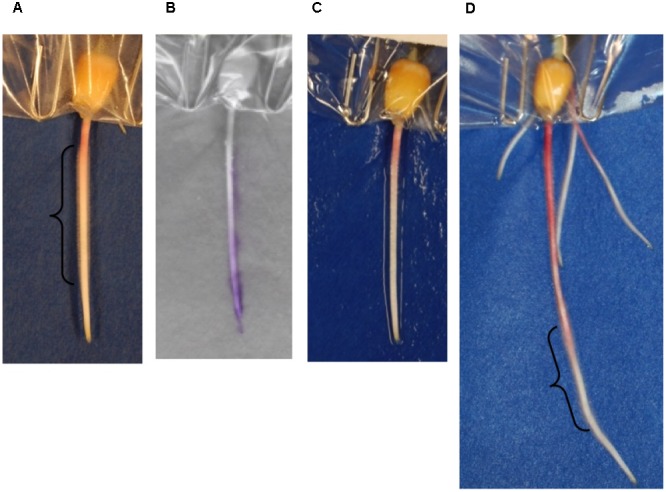
Effects of blotting ninhydrin paper on root hairs. A 3-day-old seedling root **(A)** before blotting, **(C)** after blotting, and **(B)** the overlay of the same seedling with developed ninhydrin paper. **(D)** The same root 48 h later. Regions of the root in which root hairs were observed are shown with a bracket.

In order to assess the impact of the method on root hair development, digital images taken before and after scanning were inspected. The blotting method damaged root hairs. Regions of the root containing root hairs before blotting (**Figure 6A**) no longer contain them after blotting (**Figure 6C**). However, root hairs were again found on regions of new growth within 2 days after blotting (**Figure 6D** and **Supplementary Figure S3**). While damage to root hairs contributes to some of the signal observed on the ninhydrin paper, signal was also observed in regions of the root that do not contain root hairs. For example, exudate production in the 3-day-old seedling in **Figure 6** is largely localized to the root tip and elongation zone, rather than the region in which root hairs were present (**Figure 6B**).

**FIGURE 7:**
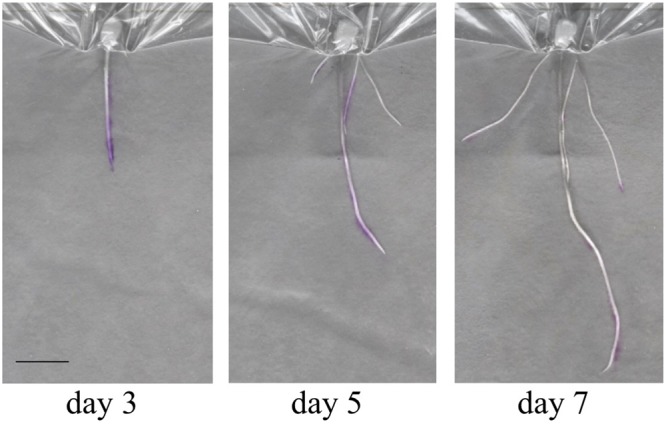
Overlay images of a seedling during the first week of growth. A single seedling during the first week of development shows production of free amines in the root tip and elongation zone (Day 3), the root base (Day 5) and at the tips of seminal roots (Day 7). Scale bar is 2 cm.

**FIGURE 8:**
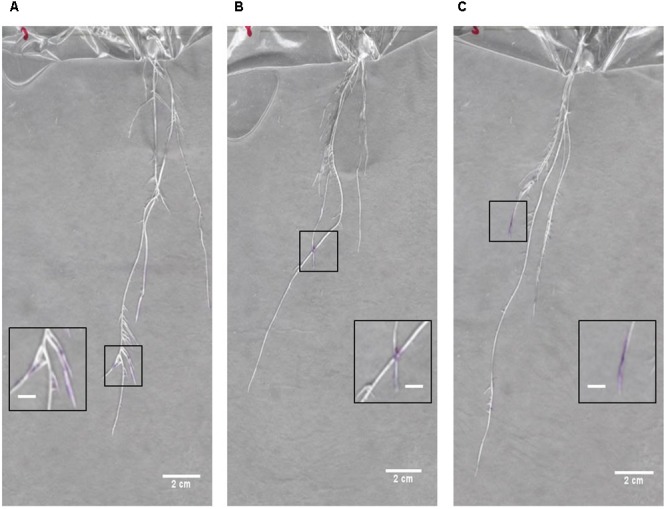
Overlay images of seedlings at 17–19 days after germination. Overlays of seedlings and developed ninhydrin paper at **(A,B)** 17 days after germination and **(C)** 19 days after germination. Insets show zoomed-in regions containing ninhydrin signal on each root system. Scale bars of main panels are 2 cm. Scale bars of inset panels are 0.5 cm.

In order to gain an initial characterization of root exudate production in young seedlings, roots growing in distilled water were blotted every other day over the course of several weeks with 3% ninhydrin paper. Grayscale images of scanned seedlings were overlaid with the corresponding developed ninhydrin paper. The first week of growth is shown for a representative B73 seedling in **Figure 7**. As expected, exudate production declined with age and was primarily localized to the apical regions of the root. By days five and seven, signal could also be observed at the base of the root and at the tip of seminal roots. Even though seedlings were grown in distilled water, signal could still be detected in some 17- and 19-day-old plants (**Figure 8**).

### Quantification of Detected Free Amines

In order to facilitate relative quantification of signal within and across papers, two 3 × 3 grids of ninhydrin were printed on the bottom corner of each ninhydrin paper (**Supplementary Presentation S1**). Directly before blotting, mock exudates of known concentrations (three replicates of three standard concentrations) were applied to one of the grids (see Materials and Methods). After scanning, the developed spots on each grid were analyzed as described in the Materials and Methods to calculate mean pixel intensity of each spot. Outlier papers were detected and removed from the dataset using a Tukey fence as described in the Section “Materials and Methods.” Overall, 20 of 30 papers collected from seedlings 3–7 days old were retained for analysis.

**FIGURE 9:**
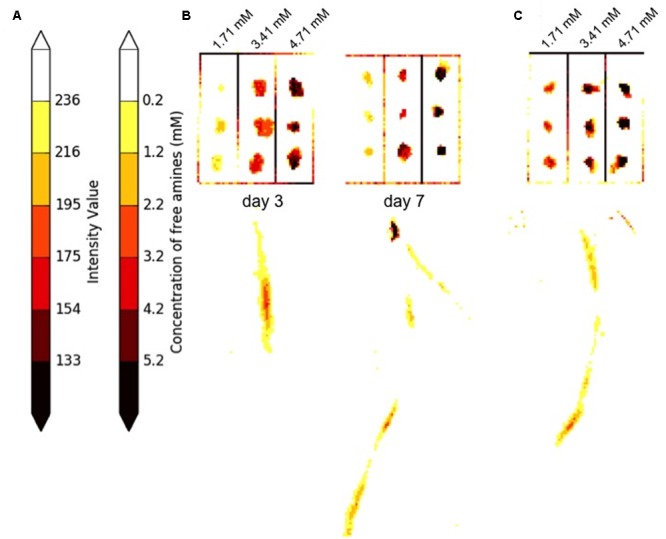
Estimation of ninhydrin signal concentration. **(A)** In 65% of developed papers, spots containing standards for a given paper align as expected to the standard scale after the heatmap is applied to the image. **(B)** Standard grids and roots from developed ninhydrin papers from the same seedling 3 and 7 days after germination. Estimation of signal concentration can be achieved by comparing regions of interest to the standard scale. **(C)** In 35% of developed papers, the colors assigned to standards within the grid of a given paper are offset from the expected color on the standard scale. A developed ninhydrin paper from a 5-day-old seedling is shown. In this case, approximate concentration of signal can be achieved by comparing the color within a region of interest to the spots on the standard grid of the same paper.

After outlier papers were removed, a linear regression of all spots for the remaining 20 papers was calculated to predict free amine concentration based on average RGB intensity. The regression was significant (*p* < 0) with an *R*^2^ of 0.72. The upper and lower 95% CIs were used to determine the range in signal intensities that could be expected at each concentration of standard. These values ranged from 19 intensity units at the lowest standard (1.71 mM) to 30 intensity units at the highest standard (4.71 mM). The slope of the regression was used to convert these ranges into concentrations, which ranged from 1.0 mM at the lowest standard to 1.6 mM at the highest standard. The average range in concentration across the three standards was 1.3 mM. This value estimates the approximate resolution in quantifying the concentration of signal on the papers. In order to visualize and compare free amine signal intensities across papers, a heatmap was applied to the grayscale images of developed ninhydrin paper using a custom Python script (**Supplementary Data Sheet S1**). The resulting visualization enabled estimation of exudate production levels within and between ninhydrin papers. The standardized color scale that was determined from the linear fit of the retained data is shown in **Figure 9A**.

Closer inspection of grids from papers retained after removal of outliers and application of the heatmap revealed that the standards for approximately 65% of papers were colored to indicate the expected concentrations on the color scale. An example of two grids displaying this pattern are shown in **Figure 9B**. The grids of standards and the corresponding root signals shown in **Figure 9B** come from blots of the same seedling at 3 and 5 days after germination. For papers in this category, regions of interest can be referenced to the standard color scale shown in **Figure 9A** for approximation of signal concentration of signal within and between papers. The data shown for the seedling in **Figure 9B** indicates that free amine production did not exceed 4.2 mM at day 3 or 7. Free amines were concentrated at a relatively apical region of the root on day 3. Signal was still observed in apical regions of the root at day 7, but the most concentrated region of signal was found more basally than was observed at day 3.

A smaller proportion of retained papers contained grids with standards that were overexposed or underexposed compared to the expected values on the color scale. An example grid and corresponding root signal from a 5-day-old seeding are shown in **Figure 9C**. The data indicates that free amine production anywhere in this root was less than 4.71 mM and that much of the production was less than 1.71 mM. The signal concentration for papers in this category can be estimated by comparing regions of interest to the grid standards found on the same paper, but these estimations should not be made by comparing signals to the standardized color scale.

## Discussion

This paper describes a cost-effective method for localization and low-resolution quantification of root exudate production during development. Roots are sensitive to chemical and physical stress, and exudate production or growth may be altered as a result of this stress, so any method developed for the purpose described here would aim to minimize such disruption (Badri and Vivanco, 2009; Baetz and Martinoia, 2014). Root hairs are recognized as important cells in production of root exudate and are estimated to make up as much as 77% of the total root surface area (Badri and Vivanco, 2009). While damage to root hairs was observed during our blotting procedure, regrowth of these cells was observed on new root tissue within 2 days (**Figure 6**). This observation and the fact that the overall root growth rate was unaffected by blotting contribute to its utility in quickly gathering information about exudate production in a living plant. The indicator ninhydrin, which reacts with free amine groups, was chosen in part because amino acids represent a major component of root exudates and also because the indicator creates a color change that is easily detected using standard light imaging (Chutipongtanate et al., 2012; Chaparro et al., 2013; Moe, 2013). These features make it possible to explore adaptation of the method to other plant species and experimental conditions. Similarly, there are many more indicators that could be suitable for use in this method, such as pH indicators (Neumann et al., 2009). A future direction will be to identify additional chemical sensors for detection of other exudate components, including sugars.

Methods have been previously developed for visualization of root exudates within the soil environment and include use of rhizotrons or rhizobox systems for in situ sampling or visualization (Haase et al., 2007; Shi et al., 2011; Oburger et al., 2013; Oburger and Schmidt, 2016). Exudates can be sampled by exposing the root/soil interface with agar containing pH indicators or complexation and chelation agents (Neumann et al., 2009). Previous studies have also described use of colorimetric indicators for visualization of exudate production (Van Egeraat, 1975; Römheld et al., 1984; Dundek et al., 2011). However, while these methods have the advantage of allowing for more precise quantification of exudates within a more natural growth environment, the method described here is generally cheaper and simpler, while minimizing exposure of the root to the visualization chemicals making it possible to collect multiple samples from one plant and to potentially collect data at higher throughput.

Total free amine concentrations have been estimated at approximately 100–300 μM in soil extracts (Jones et al., 2002; Baudoin et al., 2003). However, growing conditions, particularly hydroponic growth systems, have previously been reported to limit free amine content of maize exudates (Oburger et al., 2013). Nitrogen limitation is an additional factor limiting free amine abundance in exudate (Carvalhais et al., 2011). The described method introduced both of these limiting factors. However, our ninhydrin paper, which was capable of detecting free amines in solution with a lower concentration limit of 140 μM (**Figure 2**), enabled visualization of free-amine containing compounds on the root surface despite these potential inhibiting effects. Therefore, we expect that our method could be useful for exudates detection across a range of plant species and experimental conditions. The root tip and apical meristem of the growing root, as well as root hairs, are known to be active regions of exudate production (Jones et al., 2002; Dennis et al., 2010). This agrees with the signal patterns observed in our data both for early seedling and later seedling growth (**Figures 7–9**). However, the method developed could be used for studies to detect exudate production patterns that may differ from this established scheme.

The method we have developed describes a semi-quantitative approach for visualization of root exudate production on the root surface. Next steps will include improving quantitative measurements of exudate production and automation of the image processing tasks. Future work will also include exploring nitrogen sources that can be added to the growth media to encourage normal growth and enhance exudate production and root growth while minimizing the amount of background signal. Other colorimetric sensors will be explored to improve sensitivity and to allow for detection of other types of exudates.

## Conclusion

The described method is simple and easy to use, consumables are inexpensive, and materials are commercially available. A flatbed scanner and open source software (Image J, GIMP2, Python/OpenCV) was used for overlaying and analyzing root images. The intermittent application of the ninhydrin paper did damage root hairs, but did not prevent normal root growth.

The rapid and low-cost detection of root exudates containing free amines was achieved by utilizing a novel colorimetric paper to detect spatial and temporal changes in exudate production on the root surface through the first 2 weeks of development.

## Author Contributions

THD and TAD made substantial contributions to the study concept and design, ninhydrin paper preparation, seedling growth, image data collection and analysis, prepared figures and references, and provided relative interpretation. MK made substantial contributions to the study concept and design, ninhydrin paper preparation, image data collection process, and handled manuscript preparation including preparation of figures and references, gave intellectual contributions, improvements and revisions. AE and DT wrote Python scripts for visualizing ninhydrin signal concentrations and helped prepare the related figure. CW, AH, and ED drafted and revised the article including preparation of figures, gave intellectual contributions, improvements, and revisions. TDB conceived and supervised the research in all aspects and made significant intellectual contributions in outlining the article, article content, and critically revising the article for final submission.

## Conflict of Interest Statement

The authors declare that the research was conducted in the absence of any commercial or financial relationships that could be construed as a potential conflict of interest.

## Footnotes

1 http://www.gimp.org

## Supplementary Material

FIGURE S1 Seedling growth pouch setup. A seedling growth apparatus was constructed according to Hund et al. (2009). Seedlings were grown vertically on germination paper and a polyethylene sheet was used to maintain moisture on the sheet and to help affix the seedling to the paper surface. Seedlings were grown in distilled water. Up to 10 plants were grown together in one container. Seedling roots were shaded from light in the growth chamber by draping the containers with sheets of lightweight black fabric.

FIGURE S2 Illustration of the blotting method. Shown is a sheet of ninhydrin paper that has been laid on top of a piece of a seedling grown as described in Section “Materials and Methods.” Regions of the germination paper that do not contain seedling have been shielded using laminated paper. The tissue paper is lightly blotted to ensure contact of all root surfaces with the ninhydrin paper.

FIGURE S3 Detailed view of root hair regrowth after blotting. A detailed view of regions of the roots from **Figures 6A,D** are shown in **(A,B)**, respectively. **(A)** Well-developed root hairs of a 3-day-old seedling root that has never been blotted. **(B)** A section of the same root is shown 48 h later. The initiation of root hair development within this timeframe can be observed.

PRESENTATION S1 Printing template for ninhydrin papers.

DATA SHEET S1 Custom script for applying a heatmap to calibrated ninhydrin papers.
