# Supplementary figures and images for: A Low-Cost Imaging Method for the Temporal and Spatial Colorimetric Detection of Free Amines on Maize Root Surfaces
Source: Front Plant Sci. 2017 Aug 30;8:1513. doi: 10.3389/fpls.2017.01513 (PMC5582365; doi:10.3389/fpls.2017.01513)

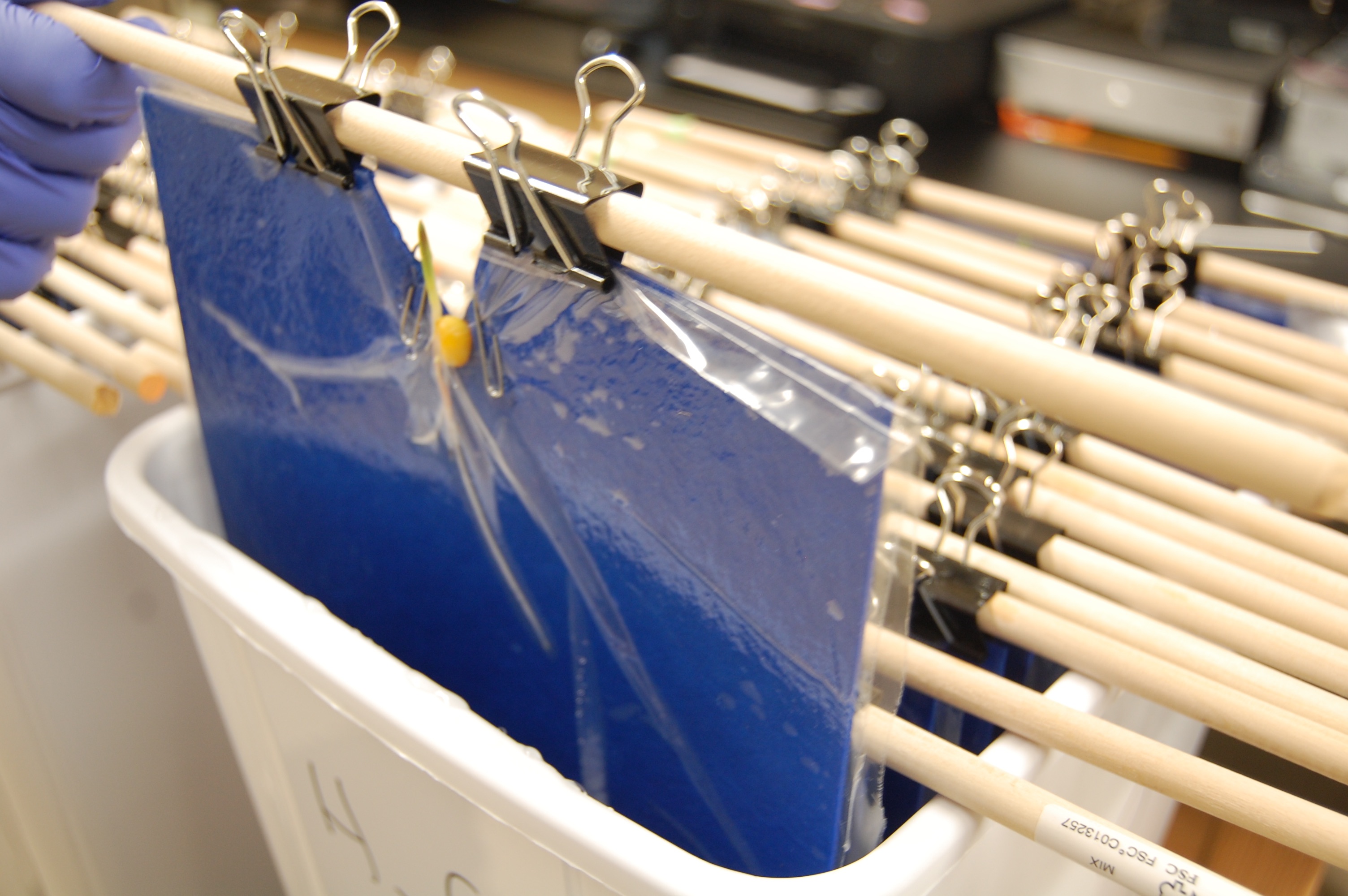

Supplement: FIGURE S1 — Seedling growth pouch setup. A seedling growth apparatus was constructed according to Hund et al. (2009). Seedlings were grown vertically on germination paper and a polyethylene sheet was used to maintain moisture on the sheet and to help affix the seedling to the paper surface. Seedlings were grown in distilled water. Up to 10 plants were grown together in one container. Seedling roots were shaded from light in the growth chamber by draping the containers with sheets of lightweight black fabric. [file Image_1.jpg]

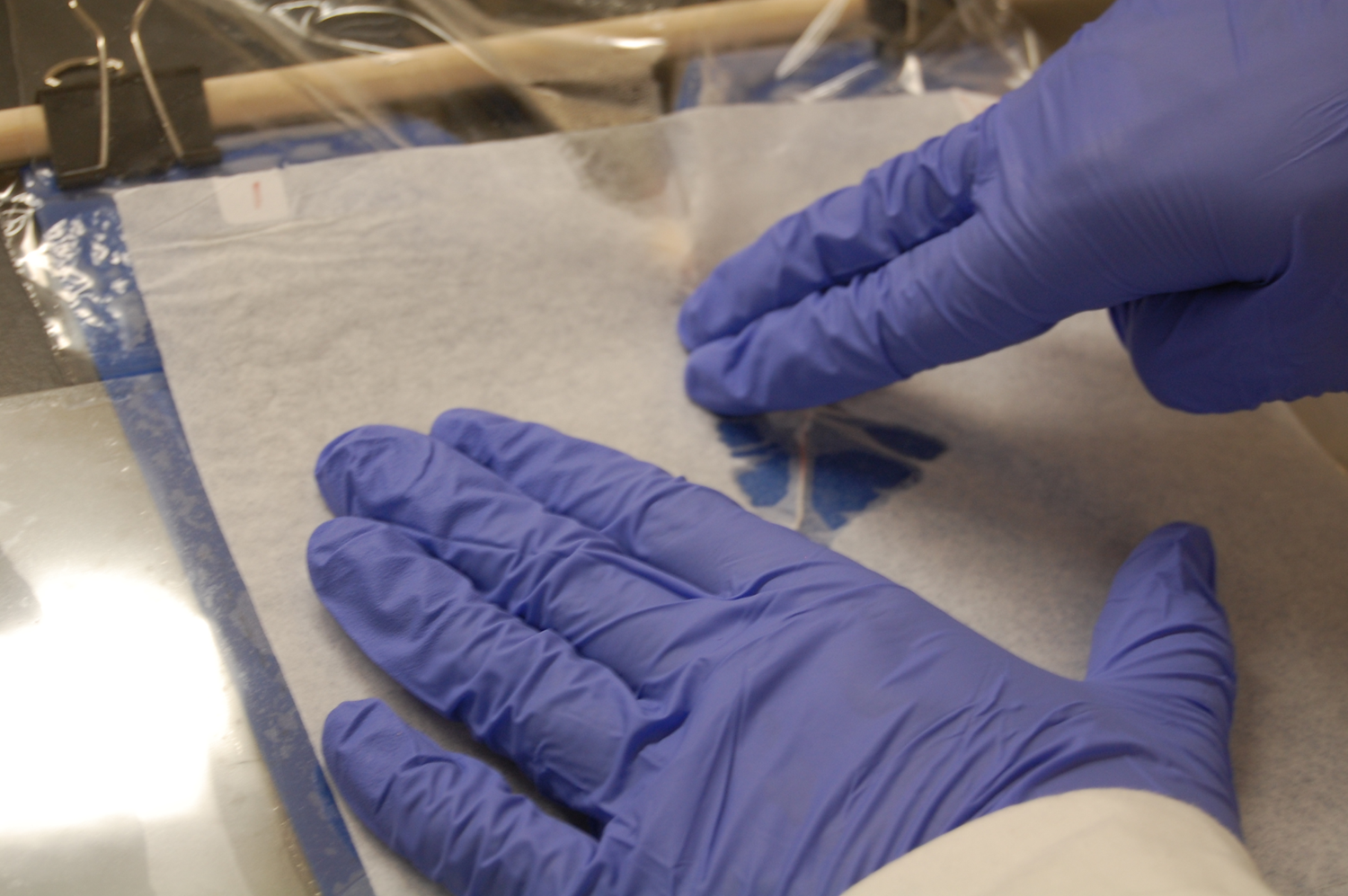

Supplement: FIGURE S2 — Illustration of the blotting method. Shown is a sheet of ninhydrin paper that has been laid on top of a piece of a seedling grown as described in Section “Materials and Methods.” Regions of the germination paper that do not contain seedling have been shielded using laminated paper. The tissue paper is lightly blotted to ensure contact of all root surfaces with the ninhydrin paper. [file Image_2.TIF]

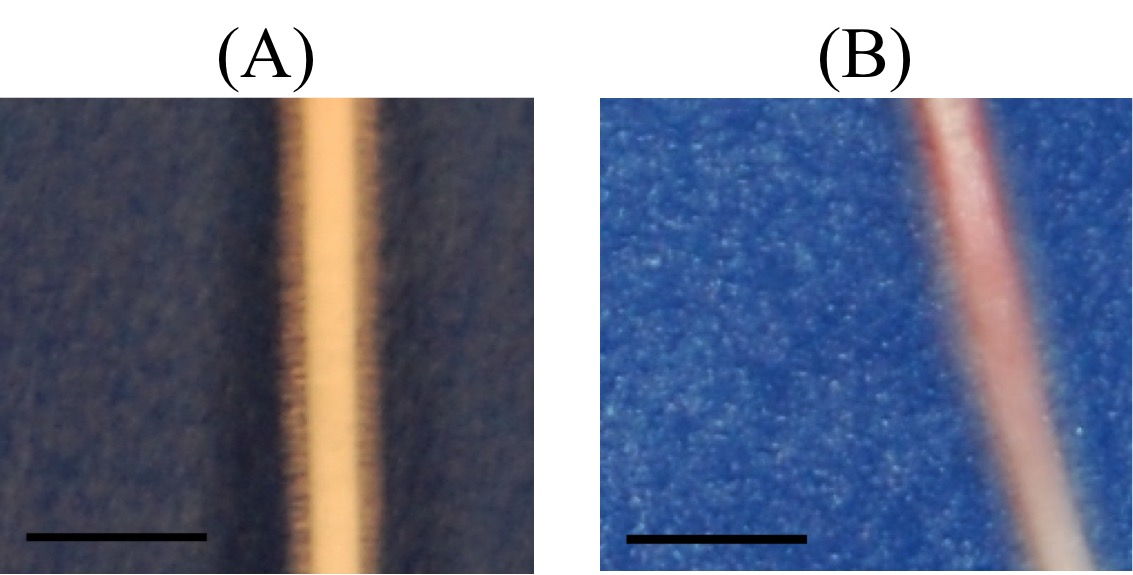

Supplement: FIGURE S3 — Detailed view of root hair regrowth after blotting. A detailed view of regions of the roots from Figures 6A,D are shown in (A,B), respectively. (A) Well-developed root hairs of a 3-day-old seedling root that has never been blotted. (B) A section of the same root is shown 48 h later. The initiation of root hair development within this timeframe can be observed. [file Image_3.TIF]

## Slide 1
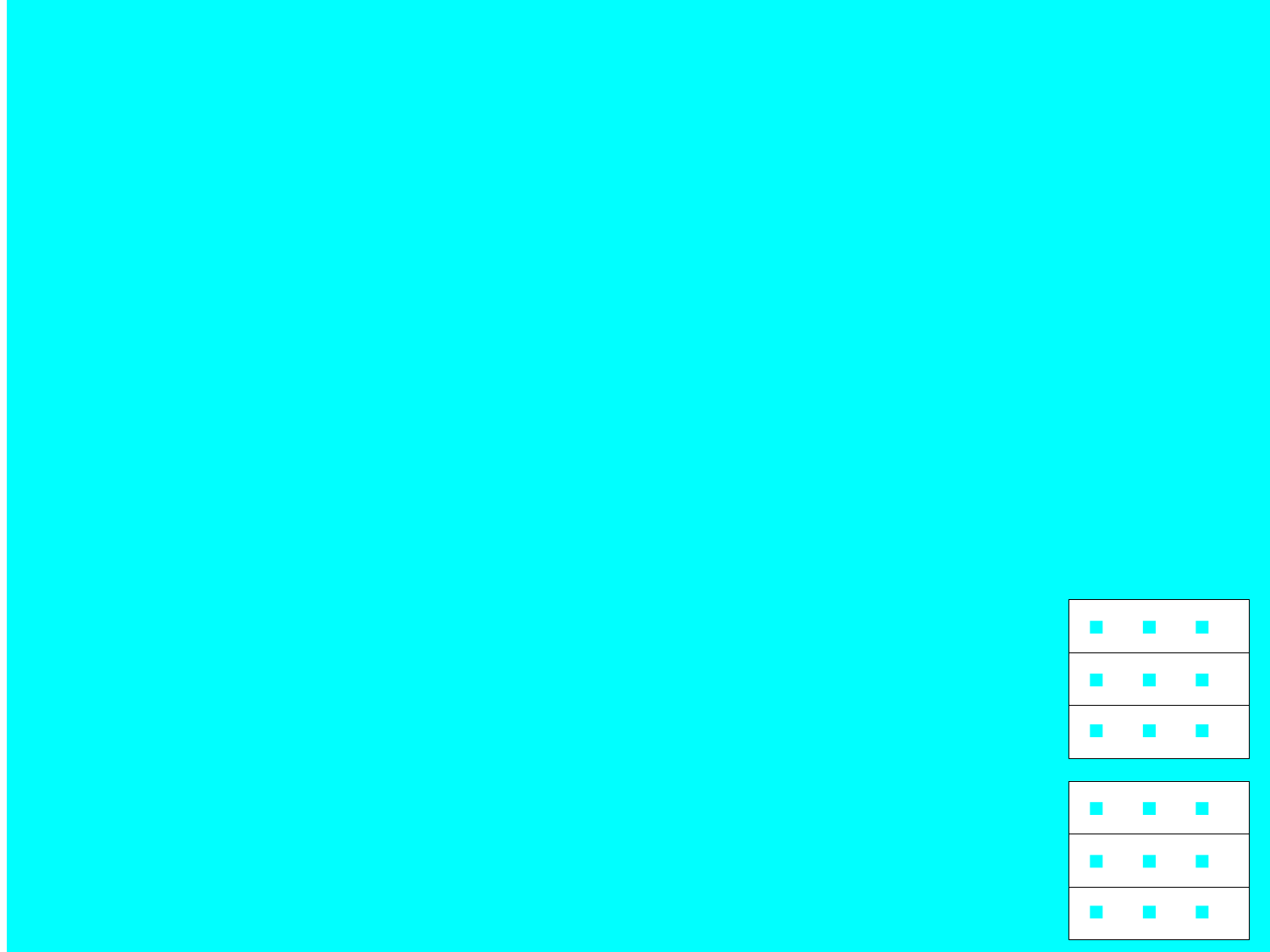

0
0

Supplement: PRESENTATION S1 — Printing template for ninhydrin papers. [file Presentation_1.PPTX]
