# Supplementary material for: A Low-Cost Imaging Method for the Temporal and Spatial Colorimetric Detection of Free Amines on Maize Root Surfaces
Source: Front Plant Sci. 2017 Aug 30;8:1513. doi: 10.3389/fpls.2017.01513 (PMC5582365; doi:10.3389/fpls.2017.01513)
Supplement: DATA SHEET S1 — Custom script for applying a heatmap to calibrated ninhydrin papers. [file Data_Sheet_1.PDF]

```
'''
##############################################################################
The purpose of this code is to create a colormap image to easily estimate the
concentration of free amines secreted by maize root. This quantification occurs
when the user compares the color of the maize root to the corresponding color
on the intensity/concentration scale.

Truc Doan, AdreAnna Ernest, Danny Tran
Edited: 7/18/2017
##############################################################################
'''

#Import libraries needed to run the code
import cv2, sys
import matplotlib.pyplot as plt
import matplotlib.ticker
import matplotlib as mpl
import matplotlib.cm as cm
import numpy as np

#Loading image with command line
filename = sys.argv[1]
img = cv2.imread(filename)

#Converting to greyscale as well as blurring the image
intensity = cv2.cvtColor(img, cv2.COLOR_BGR2GRAY)
blur = cv2.GaussianBlur(intensity,(7,7),0)

#Make plot with vertical (default) colorbar
fig, ax = plt.subplots()

#Defining colorbar template
cmap=cm.hot

#Define the number of bins between maximum and minimum pixel intensities then normalize
bounds = np.linspace(113.2521905, 256.5651239, 8)
norm = mpl.colors.BoundaryNorm(bounds, cmap.N)

#Create the colormap image
```

```
colormapImg = ax.imshow(blur, interpolation='nearest', cmap=cmap, norm=norm)

#Create two color bars - 1 for intensity and 1 for concentration
#Acknowledgement to Michael Kangas for drawedge parameter
cs = plt.colorbar(colormapImg, spacing = 'proportional', drawedges=True,
    extend='both', format = '%1i', norm=norm)
cs2 = plt.colorbar(colormapImg, spacing = 'proportional', drawedges=True,
    extend='both', format = '%1i', norm=norm)

#For Concentration colorbar (cs.)
#Defining tick labels as well as tick locations
concentration_tick = [0.2, 1.2, 2.2, 3.2, 4.2, 5.2]
tick_loc = [(concentration_tick[0]*-20.4732762) + 240.1865029,
    (concentration_tick[1]*-20.4732762) + 240.1865029,
            (concentration_tick[2]*-20.4732762) + 240.1865029,
                (concentration_tick[3]*-20.4732762) + 240.1865029,
            (concentration_tick[4]*-20.4732762) + 240.1865029,
                (concentration_tick[5]*-20.4732762) + 240.1865029]

#For pixel intensity colorbar (cs2.)
#Defining tick labels as well as tick locations
rgb_tick = [236, 216, 195, 175, 154, 133]
tick_loc2 = [236.0918477, 215.6185715, 195.1452953, 174.6720191,
    154.1987429, 133.7254667]

#Placing the tick labels according to the location provided in tick_loc
cs.locator     = matplotlib.ticker.FixedLocator(tick_loc)
cs.formatter   = matplotlib.ticker.FixedFormatter(concentration_tick)
cs.update_ticks()

#Placing the tick labels according to the location provided in tick_loc2
cs2.locator     = matplotlib.ticker.FixedLocator(tick_loc2)
cs2.formatter   = matplotlib.ticker.FixedFormatter(rgb_tick)
cs2.update_ticks()


#Adding labels
cs2.ax.set_ylabel('Intensity Value')
cs.ax.set_ylabel('Concentration of free amines (mM)')
ax.set_xlabel('X Pixel Coordinates')
plt.ylabel('Y Pixel Coordinates')

#Allowing the user to save image under the desired name
name = input('Enter the name you wish the save the file as ')

```

```
#Adjusting x and y axis to desired format
#Finally, displaying the heatmap
plt.gca().invert_yaxis()
plt.savefig(name + '.tif') #Saving image as a tif for better quality
plt.show()
```
