# Supplementary material for: A Low-Cost Imaging Method for the Temporal and Spatial Colorimetric Detection of Free Amines on Maize Root Surfaces
Source: Front Plant Sci. 2017 Aug 30;8:1513. doi: 10.3389/fpls.2017.01513 (PMC5582365; doi:10.3389/fpls.2017.01513)
Supplement: Supplementary file 6 [file Table_1.DOCX]

**Table 1:**

This table includes all VueScan software settings used for data collection of the ninhydrin tissue paper after development of root blot.

| **Input** |  |  |
| --- | --- | --- |
|  | Options: | Professional |
|  | Task: | Scan to file |
|  | Source: | PerfectionV700(scanner name) |
|  | Mode: | Flatbed |
|  | Media: | Color |
|  | Media Size: | Auto |
|  | Bits per Pixel: | 24 bit RGB |
|  | Preview Resolution: | 75 dpi |
|  | Scan Resolution: | 800 dpi |
|  | Auto Rotate: | Unchecked |
|  | Auto Flip: | Unchecked |
|  | Rotation: | None |
|  | Auto Skew: | Unchecked |
|  | Skew: | Zero |
|  | Mirror: | Checked |
|  | Auto Save: | Scan |
|  | Auto Print: | None |
|  | Auto Repeat: | None |
|  | Number of Sample: | One |
|  | Show Texture: | Unchecked |
|  | Lock image color: | Unchecked |
|  | Default folder: | Folder name where you are saving images |
|  | TIFF file name: | @.tiff |
|  | Default options: | Unchecked |
|  |  |  |
| **Crop** |  |  |
|  | Crop Size: | Maximum |
|  | Multi crop: | Off |
|  | Show multi outline: | Checked |
|  | Lock aspect ratio: | Off |
|  | Border (%): | 0 |
|  | Buffer (%): | 5 |
|  | Preview area: | Default |
|  | Default options: | Unchecked |
|  |  |  |
| **Filter** |  |  |
|  | Restore colors: | Unchecked |
|  | Restore fading: | Unchecked |
|  | Grain reduction: | None |
|  | Sharpen: | Unchecked |
|  | Descreen: | Unchecked |
|  | Flatten: | Unchecked |
|  |  |  |
| **Color** |  |  |
|  | Color balance: | Auto levels |
|  | Black point (%): | 0 |
|  | White point (%): | 1 |
|  | Curve low: | 0.25 |
|  | Curve high: | 0.61 |
|  | Brightness: | 1 |
|  | Brightness red: | 1 |
|  | Brightness green: | 1 |
|  | Brightness blue: | 1 |
|  | Scanner color space: | Default |
|  | Printer color space: | sRGB |
|  | Show IT8 outline: | Unchecked |
|  | Output color space: | Apple RGB |
|  | Monitor color space: | Apple RGB |
|  | View color: | RGB |
|  | Pixel colors: | Unchecked |
|  | Default options: | Unchecked |
|  |  |  |
| **Output** |  |  |
|  | Default folder: | Folder name where you are saving images |
|  | Printed size: | Scan size |
|  | Magnification (%): | 100 |
|  | Auto file name: | Checked |
|  | TIFF file: | Checked |
|  | TIFF file name: | @.tif |
|  | TIFF size reduction: | 1 |
|  | TIFF multi page: | Unchecked |
|  | TIFF file type: | 24 bit RGB |
|  | TIFF compression: | Auto |
|  | TIFF DNG format: | Unchecked |
|  | TIFF DNG format: | Unchecked |
|  | TIFF profile: | Checked |
|  | JPEG file: | Unchecked |
|  | PDF file: | Unchecked |
|  | OCR text file: | Unchecked |
|  | Index file: | Unchecked |
|  | Raw file: | Unchecked |
|  | Description: | Blank |
|  | Copyright: | Blank |
|  | Date: | Blank |
|  | Long file: | Checked |
|  | Log file max size (MB) | 2 |
|  | Default options: | Unchecked |
|  |  |  |
| **Prefs** |  |  |
|  | Language: | English |
|  | Font size (pt): | 12 |
|  | Crop units: | Inch |
|  | Printed units: | Inch |
|  | External viewer: | Viewer |
|  | Viewer: | Default |
|  | Auto load options: | Checked |
|  | Graph type: | Off |
|  | Button 1 action: | None |
|  | Button 2 action: | None |
|  | Button 3 action: | None |
|  | Button 4 action: | None |
|  | Display raw scan: | Checked |
|  | Splash screen: | Checked |
|  | Histogram type: | Linear |
|  | Animate crop box: | Checked |
|  | Thick crop box: | Checked |
|  | Add extensions: | Checked |
|  | Substitute date: | Checked |
|  | Warn on overwrite: | Checked |
|  | Warn on not ready: | Checked |
|  | Warn on no scanner: | Checked |
|  | Exit when done: | Unchecked |
|  | Beep when done: | 0 |
|  | Beep when auto eject: | Unchecked |
|  | Use temp file name: | Unchecked |
|  | Anti alias text: | Checked |
|  | Anti alias images: | Checked |
|  | Enable density display: | Unchecked |
|  | Enable raw from disk: | Unchecked |
|  | Enable TIFF thumbnails: | Unchecked |
|  | Disable scanners: | None |
|  | Enable sliders: | Checked |
|  | Enable spin buttons: | Checked |
|  | Enable popup tips: | Checked |
|  | Calibration period: | 30 |
|  | Image memory (MB): | 2048 |
|  | Window maximized: | Unchecked |
|  | Window iconized: | Unchecked |
|  | Window X offset: | 192 |
|  | Window Y offset: | 73 |
|  | Window X size: | 1728 |
|  | Window Y size: | 928 |
|  | Default options: | Unchecked |
